# High frequencies of Y-chromosome haplogroup O2b-SRY465 lineages in Korea: a genetic perspective on the peopling of Korea

**DOI:** 10.1186/2041-2223-2-10

**Published:** 2011-04-04

**Authors:** Soon-Hee Kim, Ki-Cheol Kim, Dong-Jik Shin, Han-Jun Jin, Kyoung-Don Kwak, Myun-Soo Han, Joon-Myong Song, Won Kim, Wook Kim

**Affiliations:** 1 School of Biological Sciences, Seoul National University, Seoul 151-747, Korea; 2 Eastern District Office, National Forensic Service, Gangwon-do 220-805, Korea; 3 Department of Biological Sciences, Dankook University, Cheonan 330-714, Korea; 4 Cardiovascular Genome Center, Yonsei University College of Medicine, Seoul 120-749, Korea; 5 Center for Genome Science, Korea National Institute of Health, Seoul 122-701, Korea; 6 DNA Analysis Division, National Forensic Service, Seoul 158-707, Korea; 7 Research Institute of Pharmaceutical Sciences and College of Pharmacy, Seoul National University, Seoul 151-742, Korea

## Abstract

**Background:**

Koreans are generally considered a Northeast Asian group, thought to be related to Altaic-language-speaking populations. However, recent findings have indicated that the peopling of Korea might have been more complex, involving dual origins from both southern and northern parts of East Asia. To understand the male lineage history of Korea, more data from informative genetic markers from Korea and its surrounding regions are necessary. In this study, 25 Y-chromosome single nucleotide polymorphism markers and 17 Y-chromosome short tandem repeat (Y-STR) loci were genotyped in 1,108 males from several populations in East Asia.

**Results:**

In general, we found East Asian populations to be characterized by male haplogroup homogeneity, showing major Y-chromosomal expansions of haplogroup O-M175 lineages. Interestingly, a high frequency (31.4%) of haplogroup O2b-SRY465 (and its sublineage) is characteristic of male Koreans, whereas the haplogroup distribution elsewhere in East Asian populations is patchy. The ages of the haplogroup O2b-SRY465 lineages (~9,900 years) and the pattern of variation within the lineages suggested an ancient origin in a nearby part of northeastern Asia, followed by an expansion in the vicinity of the Korean Peninsula. In addition, the coalescence time (~4,400 years) for the age of haplogroup O2b1-47z, and its Y-STR diversity, suggest that this lineage probably originated in Korea. Further studies with sufficiently large sample sizes to cover the vast East Asian region and using genomewide genotyping should provide further insights.

**Conclusions:**

These findings are consistent with linguistic, archaeological and historical evidence, which suggest that the direct ancestors of Koreans were proto-Koreans who inhabited the northeastern region of China and the Korean Peninsula during the Neolithic (8,000-1,000 BC) and Bronze (1,500-400 BC) Ages.

## Background

The Koreans are geographically a Northeast Asian group, who are thought to be most closely related to Altaic language-speaking populations. Anthropological and archaeological evidence suggests that the early Korean population was related to Mongolian ethnic groups, who inhabited the general area of the Altai Mountains and the Lake Baikal regions of southeastern Siberia [1]. Based on archaeological data, the earliest modern human lithic cultures date from 25,000 to 45,000 years ago in the Altai Mountains and southeastern Siberia and the Korean Peninsula [2,3]. According to Korea's founding myths, the Gojoseon (the first state-level society) was established around 2,333 BC in the region of southern Manchuria, but later stretched from the northeastern region of China to the northern part of the Korean Peninsula. Thus, the ancient Koreans (proto-Koreans) may have shared a common origin with the Northeast Asian groups who inhabited the general area of the southeastern Siberia and Manchuria during the Neolithic (8,000-1,000 BC) and Bronze (1,500-400 BC) Ages [1,4].

However, like many debates on the genetic history of human populations, the origin of the Korean population remains controversial. Studies of classic genetic markers have shown that, among the East Asians, Koreans tend to have the closest genetic affinity with Mongolians [5,6]. These findings support the first model, that a northeastern Asian origin is most likely, followed by a southeastward migration into the Korean Peninsula. The second model suggests a bi- and/or multidirectional route, with one migration through northeastern Asia and one through southeastern Asia. Recent surveys of genetic variation using two haploid markers (mitochondrial DNA and the Y chromosome) showed that the Korean population contains lineages typical of both Southeast and Northeast Asian populations [7-9]. These results led us to consider that the peopling of Korea might have involved multiple events, and that different aspects might be revealed by appropriate additional genetic markers and DNA samples.

Based on recent studies of Y-chromosome variation, the East Asian gene pool is almost completely contained within three major Y-chromosome lineages (haplogroups C, D and K) [7,10]. East Asian populations have major expansions of haplogroup O-M175 lineages (a lineage within K), although there are significant genetic differences in other lineages between the populations [8,11]. Most Y chromosomes found in the Korean population also belong to haplogroup O-M175 and its sublineages [8,9]. The Korean population has a high frequency of the haplogroup O3-M122 lineage, which is shared mainly with Chinese and Southeast Asian populations. By contrast, the haplogroup O2b-SRY465 lineages (and its sublineage) are found with high frequency and diversity specifically among modern populations of Japan and Korea [8,12,13]. These chromosomes are absent in most populations in China, but they have been detected in some samples of Beijing-Han Chinese, Manchurians, Mongolians and Southeast Siberians [7,8,14].

Hammer and Horai [12] hypothesized that the haplogroup O2b-SRY465 and its sublineage, O2b1-47z, might be Yayoi male lineages, which contributed to the contemporary mainland Japanese population via a process of demic diffusion during the Yayoi period from the Korean Peninsula, around 2,300 years ago. Hammer *et al. *[13] also suggested that the haplogroup O2b1-47z mutation arose on an ancestral O2b-SRY465 chromosome during early phases of the Yayoi migration. This lineage is also distributed sporadically in the Mongol, Manchu and Southeast Siberian populations, and in Indonesia, the Philippines, Vietnam and Micronesia [8,13,14]. Lin *et al. *[15] suggested that the O2b1-47z Y chromosome associated with the Y2 allele might have originated from an ancestral population in Henan or the southern parts of Shanxi near the Yellow River in China.

Thus, to understand the male lineage history of Korea, more data from such informative genetic markers from Korea and its surrounding regions are necessary. In the present study, 25 Y-chromosome single-nucleotide polymorphism (Y-SNP) markers and 17 Y-chromosome short tandem repeat (Y-STR) loci (Yfiler) were genotyped in 1,108 men from East Asian, several populations, to not only identify haplogroup O2b lineages and trace their migration history, but also to distinguish populations with different genetic backgrounds.

## Methods

This study was approved by the Ethics Committee and institutional review boards of Institute of Bio-Science and Technology in the Dankook University. Separate written informed consent was obtained for enrollment from all participants.

### Subjects

In this study, we analyzed 1,108 men, representatives of several East Asian populations (Korean, Japanese, Mongolian, Chinese, Indonesian, Filipino, Thai, Vietnamese; Table 1). The DNA samples included subsets of the samples examined by Kim *et al*. [16] and Jin *et al*. [8], although the exact number of subjects for each population occasionally varied between the studies. In addition, we included new Korean samples collected from 506 people residing in six major provinces in Korea [17]. DNA was prepared from whole blood taken from each participant by a standard method [18], or extracted from buccal cells as previously described [19].

**Table 1:** Sample sizes and references for East Asian populations studied (n = 1,108)

Geographic affiliation	Population (n)	Ethnic/regional group (n)	References	YHRD accession number
Northeast Asia	Korean (506)			
		Seoul/Gyeonggi (110)	Kim *et al*. (2010) [17]	YA003585
		Chungcheong (72)	Kim *et al*. (2010) [17]	YA003588
		Gangwon (63)	Kim *et al*. (2010) [17]	YA003587
		Gyeongsang (84)	Kim *et al*. (2010) [17]	YA003586
		Jeolla (90)	Kim *et al*. (2010) [17]	YA003584
		Jeju (87)	Kim *et al*. (2010) [17]	YA003583
	Japanese (157)			
		Ibaraki (50)	Kim *et al*. (2000) [16]	YA003199
		Osaka/Tokyo (6)	Kim *et al*. (2000) [16]	YA003199
		Tokushima (57)	Kim *et al*. (2000) [16]	YA003199
		Yamaguchi (44)	Kim *et al*. (2000) [16]	YA003199
	Mongolian (81)			
		Buryat (36)	Kim *et al*. (2000) [16]	YA003198
		Khalkh (45)	Jin *et al*. (2003) [8]	YA003670
	Chinese (175)			
		Beijing-Han (51)	Kim *et al*. (2000) [16]	YA003197
		Manchurian (30)	Jin *et al*. (2003) [8]	YA003195
		Xian (34)	Kim *et al*. (2000) [16]	YA003671
Southeast Asia		Yunnan-Han (60)	Jin *et al*. (2003) [8]	YA003196
	Indonesian (37)			
		Java (37)	Kim *et al*. (2000) [16]	YA003200
	Filipino (64)			
		Cebuno speak (33)	Kim *et al*. (2000) [16]	YA003202
		Davao/Mondoro (2)	Kim *et al*. (2000) [16]	YA003202
		Tagalog speak (29)	Kim *et al*. (2000) [16]	YA003202
	Thai (40)			
		Chiang Mai (32)	Kim *et al*. (2000) [16]	YA003203
		Bangkok (6)	Kim *et al*. (2000) [16]	YA003203
		Surin (2)	Kim *et al*. (2000) [16]	YA003203
	Vietnamese (48)			
		Hanoi (48)	Kim *et al*. (2000) [16]	YA003201

### Y-SNP genotyping

**Figure 1:**
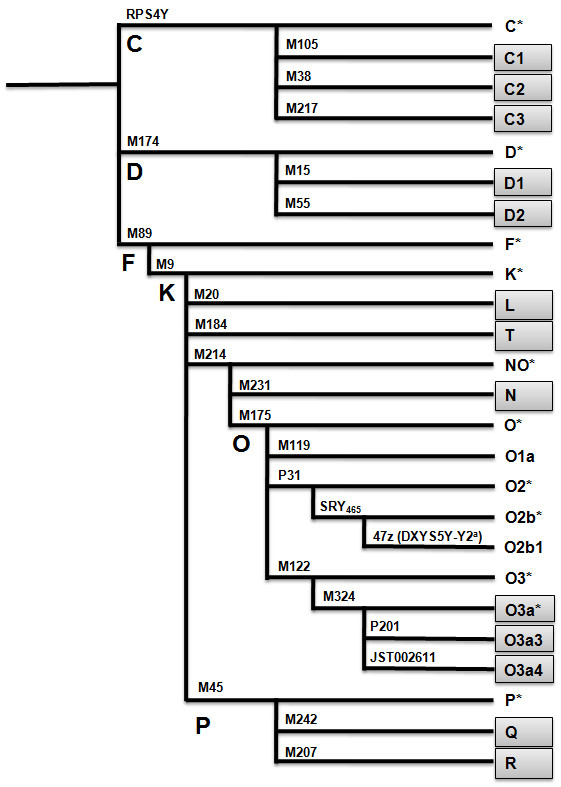
**Phylogenetic tree defined with the 25 Y-chromosome single-nucleotide polymorphisms (Y-SNPs)**. Gray squares represent an additional 13 haplogroups in this study. ^a^The O2b1 is associated with the Y2 allele [15].

Initially all the samples were analyzed for 12 Y-SNP markers (M9, M45, M89, M119, M122, M174, M175, M214, P31, SRY465, 47z and RPS4Y) using a previously described protocol [17]. The samples belonging to haplogroups C, D, K, NO, O3 and P were subjected to further typing with an additional 13 Y-SNP markers to designate the subclades: two three-plex, three two-plex and one single-plex SNaPshot assay were developed for these 13 Y-SNP markers (Figure 1). The nomenclature of the haplogroups followed that of the Y Chromosome Consortium [20]. Primers for PCR and single-base extension (SBE) reactions were designed (Primer 3.0 program; http://frodo.wi.mit.edu/primer3/, Cambridge, MA, USA) (see Additional file 1, Table S1; see Additional file 2, Table S2). Conditions of the SNaPshot assays were the same as those previously described [17], with the exception of PCR purification; in our assay, the PCR products were purified by adding 2 μl of an exonuclease I-shrimp alkaline phosphatase preparation (Exo-SAP; USB Corp., Cleveland, OH, USA) to 5 μl PCR product.

### Y-STR genotyping

Y-STRs (DYS456, DYS389I, DYS390, DYS389II, DYS458, DYS19, DYS385a/b, DYS393, DYS391, DYS439, DYS635, DYS392, YGATA H4, DYS437, DYS438 and DYS448) were amplified (AmpFlSTR Yfiler kit; Applied Biosystems, Foster City, CA, USA) and analyzed by capillary electrophoresis using a genetic analyzer (ABI Prism 3730; Applied Biosystems) and accompanying software (GeneMapper ID v3.2; Applied Biosystems). Alleles were named according to the recommendations of the International Society of Forensic Genetics [21]. The 25 Y-SNP markers and 17 Y-STR loci of the 1,108 men in this study have been submitted to the Y Chromosome Haplotype Reference Database (Table 1).

### Data analysis

The genetic differentiation between different population samples and its statistical significance were assessed via *F*_ST _(Y-SNP) values. The population genetic structure of the ethnic and/or regional groups was assessed by analysis of molecular variance (AMOVA) using the *R*_ST _(Y-STR) values [22]. The calculations of diversity indices, *F*_ST _and AMOVA were performed using Arlequin software (v3.0; http://cmpg.unibe.ch/software/arlequin3/) [23]. Population pairwise *F*_ST _values were visualized by multidimensional scaling (MDS) plot analyses (PASW Statistics 17; SPSS Inc., Cary, NC, USA). Haplotype diversity and mean number of pairwise differences of 15 Y-STR markers (excluding DYS385a/b in Yfiler) linked with the haplogroups O2b*, O2b1, O3 and C were also calculated (Arlequin 3.0 package) [23]. In addition, the genetic diversity of average variance ratio for the Y-STR markers was estimated, as described previously [24]. Median-joining (MJ) network [25] of the haplogroups O2b*, O2b1, O3 and C was constructed from the data of nine Y-STRs (NETWORK 4.5 software; http://www.fluxus-technology.com). The MJ network was constructed by weighting 99 (47z), 10 (DYS438, DYS392), nine (DYS393), eight (DYS437, DYS390), five (DYS391, DYS19), two (DYS389I) and one (DYS439) [26]. Coalescence times for subsets of chromosomes defined by the SNP markers were estimated (BATWING; http://www.mas.ncl.ac.uk/~nijw/) [27], assuming a generation time of 30 years. Locus-specific STR mutation rates (father-son mutation rates) based on previously described measurements, and various demographic models, were tested in different runs of the program [28].

## Results and discussion

**Figure 2:**
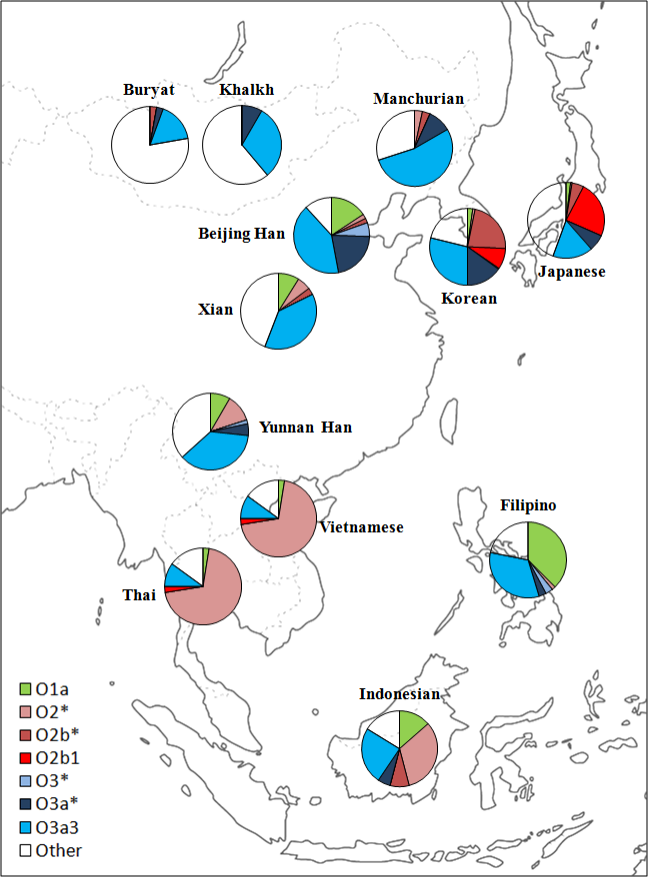
**Distribution of Y haplogroup O lineages in East Asia**. Circled areas are proportional to haplogroup frequency, and haplogroups are represented by different colors.

This survey of East Asian Y-SNPs identified 18 haplogroups, 15 of which were present in our Korean group (Table 2; see Additional file 3, Table S3). In general, we found the East Asian population groups to be characterized by male haplogroup homogeneity, showing mostly expansions of haplogroup O-M175 lineages (Figure 2). Haplogroups O3a3-P201, O2b*-SRY465, O3a*-M324, C3-M217 and O2b1-47z (a sublineage within O2b) were the most frequent found in Koreans, and accounted for 28.9, 22.5, 15.0, 12.3 and 8.9% of Korean Y chromosomes, respectively.

**Table 2:** Distribution of Y chromosome haplogroup frequencies in the East Asian populations studied

Population (n)	Samples of haplogroups in East Asian populations, n (%)																								
	
	C				D			F*	K*	L	T	NO*	N	O*	O1	O2			O3				P*	Q	R
												
	C*	C1	C2	C3	D*	D1	D2								O1a	O2*	O2b*	O2b1	O3*	O3a*	O3a3	O3a4			
**Northeast Asia**																									
Korean (506)	1 (0.20)	1 (0.20)	0	62 (12.25)	0	0	8 (1.58)	0	0	3 (0.59)	0	0	23 (4.55)	0	11 (2.17)	5 (0.99)	114 (22.53)	45 (8.89)	2 (0.40)	76 (15.02)	146 (28.85)	0	0	7 (1.38)	2 (0.40)
Japanese (157)	0	8 (5.00)	0	11 (6.88)	0	0	46 (29.30)	0	0	0	0	0	4 (2.50)	0	3 (1.88)	1 (0.63)	8 (5.10)	38 (23.75)	0	11 (6.88)	27 (16.88)	0	0	0	0
Mongolian (81)																									
Buryat (36)	0	0	0	16 (44.44)	0	1 (2.78)	0	1 (2.78)	0	0	0	0	9 (25.00)	0	0	0	1 (2.78)	0	0	1 (2.78)	6 (16.67)	0	0	0	1 (2.78)
Khalkh (45)	0	0	0	21 (46.67)	0	1 (2.22)	0	0	0	0	0	0	6 (13.33)	0	0	0	0	0	0	3 (6.67)	11 (24.44)	0	0	1 (2.22)	2 (4.44)
N.China (115)																									
Beijing-Han (51)	0	0	0	3 (5.88)	0	1 (1.96)	1 (1.96)	0	0	0	0	0	1 (1.96)	0	8 (15.69)	1 (1.96)	1 (1.96)	0	3 (5.88)	11 (21.57)	21 (41.18)	0	0	0	0
Manchurian (30)	0	0	0	8 (26.67)	0	0	0	0	0	0	0	0	0	0	0	1 (3.33)	1 (3.33)	0	0	3 (10.00)	16 (53.33)	0	0	0	1 (3.33)
Xian (34)	0	0	0	8 (23.53)	0	3 (8.82)	0	0	0	0	0	0	2 (5.88)	0	3 (8.82)	2 (5.88)	1 (2.94)	0	0	0	13 (38.24)	0	0	1 (2.94)	1 (2.94)
**Southeast Asia**																									
South China (60)																									
Yunnan-Han (60)	1 (1.67)	0	0	5 (8.33)	0	1 (1.67)	0	7 (11.67)	0	0	0	0	4 (6.67)	0	5 (8.33)	7 (11.67)	0	0	1 (1.67)	3 (5.00)	22 (36.67)	0	0	3 (5.00)	1 (1.67)
Indonesian (37)	1 (2.70)	0	0	1 (2.70)	0	0	0	1 (2.70)	0	0	0	0	0	0	5 (13.51)	12 (32.43)	3 (8.11)	0	0	2 (5.41)	9 (24.32)	0	0	2 (5.41)	1 (2.70)
Filipino (64)	1 (1.56)	0	0	1 (1.56)	0	0	0	5 (7.81)	3 (4.69)	0	0	0	0	0	24 (37.50)	1 (1.56)	0	0	2 (3.13)	2 (3.13)	21 (32.81)	0	0	2 (3.13)	2 (3.13)
Thai (40)	0	0	0	1 (2.50)	0	1 (2.50)	0	1 (2.50)	0	0	0	0	0	0	1 (2.50)	28 (70.00)	0	1 (2.50)	0	0	4 (10.00)	0	0	1 (2.50)	2 (5.00)
Vietnamese (48)	0	0	0	6 (12.50)	0	1 (2.08)	0	0	1 (2.08)	0	0	0	1 (2.08)	0	2 (4.17)	5 (10.42)	2 (4.17)	2 (4.17)	1 (2.08)	14 (29.17)	13 (27.08)	0	0	0	0

Total (1,108)	4 (0.36)	9 (0.81)	0	143 (12.87)	0	9 (0.81)	55 (4.96)	15 (1.35)	4 (0.36)	3 (0.27)	0	0	50 (4.50)	0	62 (5.58)	63 (5.67)	131 (11.82)	86 (7.74)	9 (0.81)	126 (11.34)	309 (27.81)	0	0	17 (1.53)	13 (1.17)

**Figure 3:**
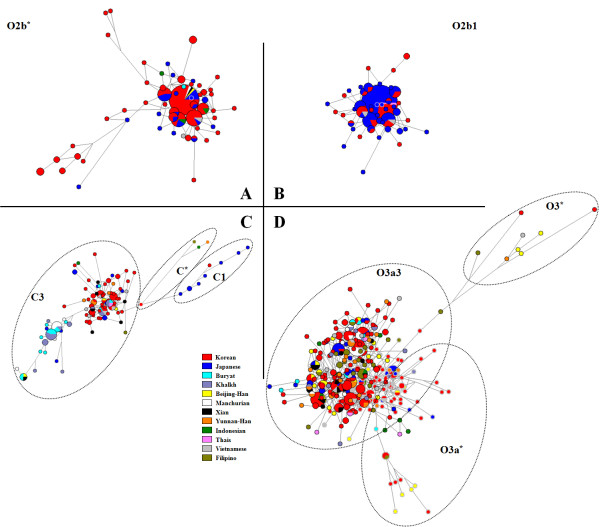
**Median-joining network for East Asian Y haplogroup lineages based on the minimum nine Y-chromosome short tandem repeat (Y-STR) haplotypes**. **(A) **Network of haplogroup O2b*; **(B) **network of haplogroup O2b1; **(C) **network of haplogroup C; **(D) **network of haplogroup O3. Circled areas are proportional to haplotype frequency; lines represent the mutational differences between haplotypes.

O3-M122 was the commonest Chinese Y-chromosome haplogroup found, and its presence in Korea may originate from demic diffusion by way of south-to-north migration [9,29,30]. C-RPS4Y was the commonest Mongolian Y-chromosome haplogroup (C3 lineage), and this is shared primarily with populations in northern Asia (including Koreans) [9,31,32]. Our network and diversity analyses of C-RPS4Y and O3 may support a southern origin that migrated into East Asia via the southern route [10,29] (Figure 3, Table 3). Interestingly, our Japanese group seemed to have both C1-M105 and C3-M217 chromosomes, whereas haplogroup C1-M105 was not present in most East Asian populations (except for Koreans), consistent with the previous report of Hammer *et al*. [13]. Haplogroup D2-M55 was found at high frequency only in Japanese subjects (29.3%), including 1.6% of Koreans, whereas it was absent elsewhere in these East Asian populations, except for the Beijing-Han group. Haplogroup D1-M15 was present at extremely low frequencies in the other East Asian populations. The occurrence of C1 and D2 chromosomes in Korea may be considered concordant with the historical views about the recent connection between the Korean and Japanese populations.

**Table 3:** 15 Y-STRs diversity of haplogroups C and O3 in East Asian populations

**Population**	**Diversity parameters**							
	
	**Haplogroup C**				**Haplogroup O3**			
		
	**n**	**Haplotype diversity**	**Mean number of pairwise differences**	**Allele size variation ratio**	**n**	**Haplotype diversity**	**Mean number of pairwise differences**	**Allele size variation ratio**
		
**NorthEast Asia**								
Korean	63	0.99 ± 0.00	5.89 ± 2.85	0.45	222	0.99 ± 0.00	8.48 ± 3.94	0.74
Japanese	15	1.00 ± 0.02	7.17 ± 3.56	0.86	38	0.99 ± 0.01	8.53 ± 4.03	0.69
Mongolian								
Buryat	14	0.97 ± 0.04	5.69 ± 2.90	0.35	7	1.00 ± 0.08	8.52 ± 4.49	0.67
Khalkh	18	0.92 ± 0.05	4.81 ± 2.46	0.28	14	1.00 ± 0.03	8.98 ± 4.40	0.80
North China								
Beijing-Han	3	1.00 ± 0.27	8.00 ± 5.13	0.60	34	1.00 ± 0.01	8.89 ± 4.20	0.79
Manchurian	7	0.52 ± 0.21	2.57 ± 1.56	0.16	19	0.95 ± 0.04	5.99 ± 2.99	0.49
Xian	7	1.00 ± 0.08	6.86 ± 3.68	0.47	13	1.00 ± 0.03	8.10 ± 4.02	0.64
**SouthEast Asia**								
South China								
Yunnan-Han	6	0.93 ± 0.12	6.47 ± 3.57	0.58	26	1.00 ± 0.01	8.53 ± 4.07	0.72
Indonesian	2	1.00 ± 0.50	10.00 ± 7.42	0.84	11	1.00 ± 0.04	8.33 ± 4.18	0.80
Filipino	2	1.00 ± 0.50	11.00 ± 8.12	1.42	26	0.99 ± 0.01	7.49 ± 3.62	0.66
Thai	1	1.00 ± 0.00	0.00 ± 0.00	-	4	1.00 ± 0.18	8.83 ± 5.17	0.87
Vietnamese	6	1.00 ± 0.10	6.60 ± 3.63	0.58	27	0.98 ± 0.02	7.98 ± 3.83	0.64

**Figure 4:**
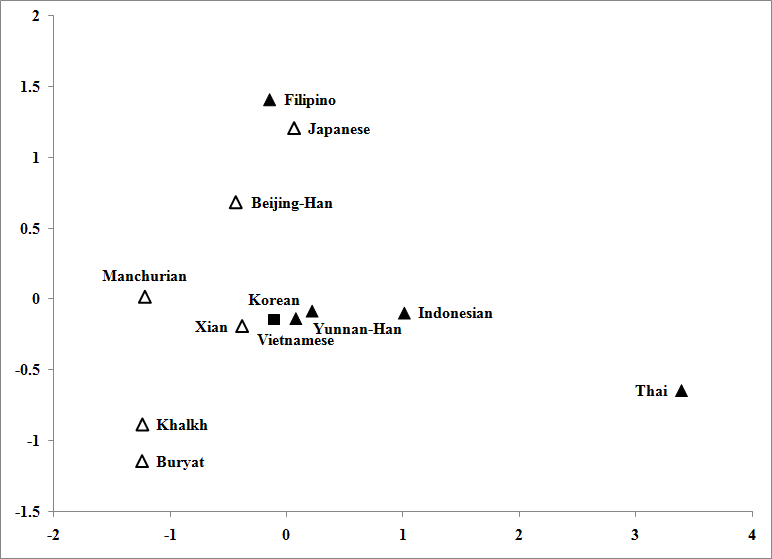
**Multidimensional scaling (MDS) plot based on *F***_**ST **_**distances of 12 populations in East Asia (see Additional file **4**, Table S4)**. Square = Koreans, open triangles = Northeast Asians, closed triangles = Southeast Asians.

Haplogroup N-M231 was present in the Korean and Mongolian groups at moderate frequencies, suggesting that the early Korean population may have shared a common origin with Mongolian ethnic groups who inhabited the general area of the Altai Mountains and Lake Baikal regions of southeastern Siberia [1]. The K-M9 defined chromosomes (L, Q and R subtypes) were also found at low frequencies in the Korean group, and these mainly occur in central and south Asia [13,33]. The presence of these haplogroups in the Korean population implies that the peopling of Korea probably involved multiple events [8,9]. Based on the result of the MDS plot (Figure 4 and Additional file 4, Table S4), the Korean population contains lineages from both the southern and northern areas of East Asia.

The geographical distribution of the O2b*-SRY465 (and its sublineage; together designated O2b-SRY465) in East Asia is shown in Figure 2. Interestingly, we found the high frequency of haplogroup O2b*-SRY465 to be characteristic of Koreans, (22.5%), but its distribution elsewhere in the East Asian populations was very patchy (Table 2, Figure 2). The cluster pattern in the O2b*-SRY465 network (Figure 3) was indicative of a single origin, although people with haplogroup O2b*-SRY465 were found to be distributed widely across both northeastern and southeastern Asia. The genetic differences between the Korean and other East Asian populations were analyzed by AMOVA (Table 4). When samples were grouped into Southeast Asians (SEA) and Northeast Asians (NEA), AMOVA could not distinguish between them. In addition, Koreans were again not distinct from either SEA or NEA, indicating that a southern versus a northern origin could not be distinguished from the Y-STR based comparison (Table 4).

**Table 4:** Results from analysis of molecular variance for 15 Y-chromosome short tandem repeats (excluding DYS385a/b in Yfiler) in East Asian populations

	Variance, % (*P *value)		
	
Grouping	Between groups	Between populations, within groups	Within populations
Korean versus SEA^a ^versus NEA^b^	-1.42 (0.86)	2.83 (0.18)	98.59 (0.06)
Korean versus SEA	-2.58 (0.71)	3.55 (0.50)	99.04 (0.22)
Korean versus NEA	-3.18 (0.34)	6.02 (0.21)	97.16 (0.03)
SEA versus NEA	-0.14 (0.54)	0.62 (0.17)	99.52 (0.17)

^a^Southeast Asian: Chinese (Yunnan-Han), Indonesian, Filipino, Thai, Vietnamese.

^b^Northeast Asian: Korean, Japanese, Chinese (Beijing-Han, Manchurian, Mongolian, Xian).

Previous studies suggested a southeastern Asian origin for O2b-SRY465 [8,13], because the entire haplogroup O in East Asia has been proposed to have a southeastern Asian origin [10]. Under the southern origin hypothesis of the Y chromosome in Asia, no extensive bottleneck or genetic isolation are expected in far-east Asia, because there is no obvious barrier except perhaps a linguistic one (Sino-Tibetan-speaking people versus Altaic-speaking people) [34]. The very low incidence of the O2b*-SRY465 haplogroup in our Chinese population group is a substantial departure from the southern-expansion hypothesis of O2b*-SRY465, and indicates that O2b*-SRY465 has undergone apparent long-term isolation in far-east Asia. Therefore, it is unlikely that southeastern Asia is the place of the early settlement of modern humans carrying O2b chromosomes. In contrast to some other sublineages within haplogroup O, the diversity of O2b*-SRY465 calculated in a previous study [13] indicated that northern populations in East Asia are more polymorphic than southern populations, implying a northern Asian origin of O2b*-SRY465, in accordance with its highest frequency distribution pattern in far-east Asia [14,35]. However, as to the issue of whether haplotype originated in prehistoric Korea or in the Japanese Archipelago within northeastern Asia, the most likely region can again be identified on the basis of the highest frequency and the highest diversity. Although there was an expected error due to small sample size, the Y-STR diversities (for example, mean number of pairwise differences and average variance ratio) were higher in Koreans than in Japanese (Table 5), consistent with previous results [13]. Both the haplogroup diversity and haplogroup frequency of O2b*-SRY465 thus suggest its early settlement in prehistoric Korea.

**Table 5:** 10 Diversity of Y-chromosome short tandem repeats in haplogroup O2b* and O2b1 in Korean and Japanese populations

**Diversity parameters**^**a**^	Haplogroup O2b*			**Haplogroup O2b1**		
	**Population (number of samples)**			**Population (number of samples)**		
		
	**Korean (114)**^**b**^	**Japanese (8)**^**b**^	**Japanese (22)**^**c**^	**Korean (45)**^**b**^	**Japanese (37)**^**b**^	**Japanese (66)**^**c**^
						
Haplotype diversity, mean ± SD	0.91 ± 0.02	0.96 ± 0.08	0.97 ± 0.02	0.91 ± 0.04	0.92 ± 0.03	0.91 ± 0.03
Number of pairwise differences, mean ± SD	2.82 ± 1.50	1.89 ± 1.20	2.91 ± 1.59	2.30 ± 1.29	2.06 ± 1.18	2.01 ± 1.15
Allele size variation ratio	0.24	0.12	0.30	0.25	0.15	0.17

^a^Indices were generated from 10 Y-chromosome short tandem repeat markers (excluding DYS385a/b, DYS448, DYS456, DYS458, DYS635 and GATA from the Yfiler system) to be consistent with Nonaka *et al*. (2007).

^b^Present study

^c^Nonaka *et al*. (2007)

Age estimates based on our Y-STR data provide further support for the initial expansion of the O2b*-SRY465 in prehistoric Korea, in accordance with the proto-Korean lineages. Based on the Korean demographic and population history, a constant population in three different BATWING models may give the best fit to our data. Thus, time to most recent common ancestor (TMRCA) for the O2b*-SRY465 lineages was 9,900 years, assuming constant population size (Table 6). According to the three different population expansion models, TMRCA within the Korean and Japanese populations and the whole of East Asia ranged from 6,000-10,000 years ago. This date corresponds to the early Neolithic Age in Korea. Therefore, the age of the O2b*-SRY465 and pattern of variation within the lineages suggests a Neolithic proto-Korean founder in a nearby part of northeastern Asia, followed by a population increase in the vicinity of the Korean Peninsula.

**Table 6:** BATWING estimates

	**Korean**		**Japanese**		**East Asian**	
			
	**O2b**	**O2b1**	**O2b**	**O2b1**	**O2b**	**O2b1**
						
Constant size	9,949 (4,598-20,408)	4,407 (1,928-10,514)	5,912 (2,438-13,800)	4,143 (1,725-9,279)	9,804 (4,751-18,489)	4,882 (2,592-8,834)
Always expanding	7,767 (3,838-17,263)	4,520 (2,198-9,281)	6,106 (2,324-15,051)	4,891 (1,840-11,906)	8,165 (3,805-16,361)	5,130 (2,553-10,152)
Constant then expanding	9,541 (4,490-19,404)	4,851 (2,493-9,394)	6,707 (1,195-27,981)	5,347 (924-22,288)	10,479 (3,922-27,984)	5,943 (1,997-17,010)

Interestingly, the O2b1-47z sublineage seems to have diverged about 4,400 years ago (Table 6) rather than in the Yayoi period, consistent with a previous estimate of 4,000 years ago [13]. Therefore, the present data support the possibility of an ancient Korean origin of O2b1-47z, rather than a Japanese origin [13]. Although O2b1-47z is at its highest haplogroup frequency in the Japanese population, the Y-STR data reveal more diversity of O2b1-47z haplotypes in Koreans, as shown by the mean number of pairwise differences and allele size variation ratio (Table 5), supporting an origin of the O2b1-47z mutation in prehistoric Korea. The Japanese samples studied here were derived from Kyushu, Shikoku and southern Honshu (the region closest to Korea), implying that the high frequency of the O2b1 lineage in Japan may be explained by genetic drift [12,16]. This finding is concordant with a previous report of Nonaka *et al. *[36], showing less diversity in Japan than in Korea (Table 5). TMRCA of O2b1-47z, and a star-cluster pattern in this study (Figure 3) and a previous study [13], all suggest the possible association of O2b1-47z with the peopling of Korea. However, because most of the Japanese O2b*-SRY465 and O2b1-47z samples were also in the core (or close to) of the cluster (Figure 3), it cannot exclude the possibility that the Japanese and the Koreans derive from the same proto-population outside of Korea (carrying these lineages) at roughly the same time. Thus, further studies of sufficient sampling in the vast East Asian region and genomewide genotyping should provide further insights.

## Conclusions

Our results support the idea that both haplogroups O2b*-SRY465 and O2b1-47z had an *in situ *origin among Northeast Asians, particularly among the prehistoric Koreans, rather than in southeast Asia or Japan as previously envisaged. The combination of the O2b initial settlement (which became an indigenous proto-Korean component) in part with the relatively recent O3 and C3 lineages (which include a Chinese component) explains some of the main events formulating the current Y chromosome composition of the Korean population. Thus, our findings are consistent with linguistic, archaeological and historical evidence, which suggest that the direct ancestors of Koreans were proto-Koreans who inhabited the northeastern region of China and the Korean Peninsula during the Neolithic (8,000-1,000 BC) and Bronze (1,500-400 BC) Ages.

## Competing interests

The authors declare that they have no competing interests.

## Authors' contributions

SHK, WNK and WKK conceived and designed the experiments; SHK, KCK, DJS, HJJ and KDK performed the experiments:. SHK, KCK, WNK and WKK analyzed the data;. MSH, JMS, WNK and WKK contributed reagents, materials and analysis tools; and SHK, MSH, WNK and WKK wrote the paper. All authors read and approved the final manuscript.

## Supplementary Material

Additional file 1 **Table S1**. Thirteen Y-chromosome single-nucleotide polymorphism (Y-SNP) markers and primer information for PCR amplification. Click here for file

Additional file 2 **Table S2**. SBE primers for the detection of 13 Y-chromosome single-nucleotide polymorphisms (Y-SNPs) studied. Click here for file

Additional file 3 **Table S3**. Haplotypes for 25 Y-chromosome single-nucleotide polymorphisms (Y-SNPs) and 17 Y-chromosome short tandem repeat (Y-STR) loci in 1,108 males from East Asian populations. Click here for file

Additional file 4 **Table S4**. *F*_ST _distances from haplogroup frequencies in East Asian populations (non-significant values are underlined). Click here for file
