# Supplementary material for: High frequencies of Y-chromosome haplogroup O2b-SRY465 lineages in Korea: a genetic perspective on the peopling of Korea
Source: Investig Genet. 2011 Apr 4;2:10. doi: 10.1186/2041-2223-2-10 (PMC3087676; doi:10.1186/2041-2223-2-10)
Supplement: Additional file 1 — Table S1. Thirteen Y-chromosome single-nucleotide polymorphism (Y-SNP) markers and primer information for PCR amplification. [file 2041-2223-2-10-S1.PDF]

Table S1. Thirteen Y-SNP markers and primer information for PCR amplification

SNP marker	Haplogroup	Mutation	Primers (5'→3')	μM	PCR size (bp)	PCR system
M105	C1	C→T	F: TGACAGTATCAAAGAAAGAAAGAGG	1.0	138	3-plex
			R: CTCGCTTTTCCACCTACTCC	1.0		
M38	C2	T→G	F: TCCCTCTGGCACTAGCAGTT	1.0	143	
			R: CACAGGGGAATGCTTACTGA	1.0		
M217	C3	A→C	F: GGAGAATGAAAAAGTTGGGTGA	1.0	135	
			R: CCATTGGAATTTAAGTGGCTTT	1.0		
M15	D1	-9bp	F: GAGGGTCTGCTAACCCACTG	1.0	133	2-plex
			R: TTGACTCTGTCCCTGCTTCA	1.0		
M55	D2	T→C	F: ACAAATAGGTGGGGCAAGAG	1.0	132	
			R: TCTGAATCCTAATGGCTGTTTTT	1.0		
M20	L	A→G	F : AGTGCTTAGCTGGGCAATTT	1.0	142	2-plex
			R : TCAGTGCAAATGCAACCATC	1.0		
M184	T	G→A	F : CAAAATGGCAAATGTGAAACA	1.0	138	
			R : TTGCCTTCTCCAAGTTTTGC	1.0		
M231	N	G→A	F : TGGAAAATGTGGGCTCGTT	1.0	120	single plex
			R : TGACGATCTTTCCCCCAAT	1.0		
M324	O3a	G→C	F: TTGAGCCCTGGAATACTAAGC	0.5	133	3-plex
			R: GATACATGGGCTGCAACAAG	0.5		
P201	O3a3	T→C	F: GTGCTGTGCAAGTTGTGTGA	0.5	149	
			R: AACCCCAAATCCCAAGGTAG	0.5		
JST002611	O3a4	C→T	F: CCAATGACCCTTTGCAGTG	0.5	150	
			R: GCCCAGATACCCAGCAGTA	0.5		
M242	Q	C→T	F : AAAAAGGTGACCAAGGTGCT	1.0	149	2-plex
			R : TTTCGCTTTAAGGGCTTTCA	1.0		
M207	R	A→G	F : GGGGCAAATGTAAGTCAAGC	1.0	124	
			R : GCTGAAGGAAAAGTGGAGTCTG	1.0		
