# Supplementary material for: High frequencies of Y-chromosome haplogroup O2b-SRY465 lineages in Korea: a genetic perspective on the peopling of Korea
Source: Investig Genet. 2011 Apr 4;2:10. doi: 10.1186/2041-2223-2-10 (PMC3087676; doi:10.1186/2041-2223-2-10)
Supplement: Additional file 2 — Table S2. SBE primers for the detection of 13 Y-chromosome single-nucleotide polymorphisms (Y-SNPs) studied. [file 2041-2223-2-10-S2.PDF]

Table S2. SBE primers for the detection of 13 Y-SNPs studied

SNP marker	Primers (5'→3')	µM	Size (bp)	SNP detected	Detection orientation	SNaPshot system
C1-M105	CAAAGAACACGTTATTACCCATA	0.4	23	A/G	R	
C2-M38	CTGACAACTTATTATGGAAAACCAACT	0.4	27	T/G	F	3-plex
C3-M217	AGTCTGACAAATGAAAAAGTTGGGTGACAC	0.4	30	A/C	F	
D1-M15	CCACTGCACCTAGGGAGACA	0.4	20	G/T	F	2-plex
D2-M55	GACAAACTGGATGACTGATGAAAAT	0.4	25	A/G	R	
L-M20	ACCAACTGTGGATTGAAAAT	0.4	20	A/G	F	2-plex
T-M184	TGACAACAGCTGTAGAAAAATTACGA	0.4	26	A/G	F	
N-N231	TTACTGTTTCTACTGCTTTC	0.4	20	A/G	F	single plex
O3a-M324	TGATCTACCTGCCCTTTCCT	0.4	20	G/C	F	
O3a3-P201	CTGACAAGTGAGAGCCAGTTAAAGCCC	0.4	27	A/G	R	3-plex
O3a4-JST002611	AGTCTGACAACGTGGTGCCTACTAGCAGGG	0.4	30	C/T	F	
Q-M242	AAAAAGGTGACCAAGGTGCT	0.4	20	C/T	F	2-ple
R-M207	TGACAATAAGTCAAGCAAGAAATTTA	0.4	26	A/G	F	

Neutral sequences are underlined
