# Supplementary material for: High frequencies of Y-chromosome haplogroup O2b-SRY465 lineages in Korea: a genetic perspective on the peopling of Korea
Source: Investig Genet. 2011 Apr 4;2:10. doi: 10.1186/2041-2223-2-10 (PMC3087676; doi:10.1186/2041-2223-2-10)
Supplement: Additional file 4 — Table S4. FST distances from haplogroup frequencies in East Asian populations (non-significant values are underlined). [file 2041-2223-2-10-S4.PDF]

Table S4. F_{ST} distances from haplogroup frequencies in 12 east Asian populations (Non-significant values are underlined)

	1	2	3	4	5	6	7	8	9	10	11	12
1 Korean	0.00000											
2 Japanese	0.08382	0.00000										
3 Beijing-Han	0.04619	0.11946	0.00000									
4 Manchurian	0.06024	0.15466	0.04215	0.00000								
5 Buryat	0.11011	0.16478	0.16295	0.12262	0.00000							
6 Khalkh	0.09855	0.16113	0.13444	0.07228	-0.00839	0.00000						
7 Xian	0.04702	0.11599	0.03627	0.00585	0.06193	0.03889	0.00000					
8 Yunnan-Han	0.05353	0.11102	0.02284	0.03873	0.11320	0.09977	0.00752	0.00000				
9 Indonesian	0.08025	0.13187	0.07359	0.12219	0.17576	0.16742	0.06271	0.02820	0.00000			
10 Filipino	0.11409	0.15839	0.04815	0.13631	0.20779	0.19352	0.07450	0.05144	0.08291	0.00000		
11 Thai	0.26795	0.28912	0.31698	0.37131	0.36552	0.36265	0.28678	0.22544	0.10361	0.32233	0.00000	
12 Vietnamese	0.02911	0.09904	0.01414	0.05943	0.12004	0.09766	0.04881	0.03666	0.05428	0.10669	0.25067	0.00000
